# Solid fuels for cooking and tobacco use and risk of major chronic liver disease mortality: a prospective cohort study of 0.5 million Chinese adults

**DOI:** 10.1093/ije/dyz216

**Published:** 2019-10-25

**Authors:** Ka Hung Chan, Derrick A Bennett, Om P Kurmi, Ling Yang, Yiping Chen, Jun Lv, Yu Guo, Zheng Bian, Canqing Yu, Xiaofang Chen, Caixia Dong, Liming Li, Zhengming Chen, Kin Bong Hubert Lam, on behalf of the China Kadoorie Biobank Study Group

**Affiliations:** 1 Clinical Trial Service Unit and Epidemiological Studies Unit, Nuffield Department of Population Health, University of Oxford, Oxford, UK; 2 Population Health Research Institute, McMaster University, Hamilton, ON, Canada; 3 MRC Population Health Research Unit, Nuffield Department of Population Health, University of Oxford, Oxford, UK; 4 Department of Epidemiology and Biostatistics, School of Public Health, Peking University Health Science Center, Beijing, China; 5 Chinese Academy of Medical Sciences, Beijing, China; 6 NCDs Prevention and Control Department, Pengzhou Center for Disease Control and Prevention, Sichuan, China; 7 NCDs Prevention and Control Department, Gansu Center for Disease Control and Prevention, Gansu, China

**Keywords:** Household air pollution, solid fuels, smoking, chronic liver disease, cohort study, China

## Abstract

**Background:**

Harmful substances in solid fuel and tobacco smoke are believed to enter the bloodstream via inhalation and to be metabolized in the liver, leading to chronic liver damage. However, little is known about the independent and joint effects of solid fuel use and smoking on risks of chronic liver disease (CLD) mortality.

**Methods:**

During 2004–08, **∼**0.5 million adults aged 30–79 years were recruited from 10 areas across China. During a 10-year median follow-up, 2461 CLD deaths were recorded. Multivariable Cox regression yielded adjusted hazard ratios (HRs) and 95% confidence intervals (CIs) for the individual associations of self-reported long-term cooking fuel and tobacco use with major CLD death.

**Results:**

Overall, 49% reported solid fuel use and 26% smoked regularly. Long-term solid fuel use for cooking and current smoking were associated with higher risks of CLD deaths, with adjusted HRs of 1.26 (95% CI, 1.02–1.56) and 1.28 (1.13–1.44), respectively. Compared with never-smoking clean fuel users, the HRs were 1.41 (1.10–1.82) in never-smoking solid fuel users, 1.55 (1.17–2.06) in regular-smoking clean fuel users and 1.71 (1.32–2.20) in regular-smoking solid fuels users. Individuals who had switched from solid to clean fuels (1.07, 0.90–1.29; for median 14 years) and ex-regular smokers who stopped for non-medical reasons (1.16, 0.95–1.43; for median 10 years) had no evidence of excess risk of CLD deaths compared with clean fuel users and never-regular smokers, respectively.

**Conclusions:**

Among Chinese adults, long-term solid fuel use for cooking and smoking were each independently associated with higher risks of CLD deaths. Individuals who had stopped using solid fuels or smoking had lower risks.

## Introduction

**Key Messages**

- Long-term solid fuel use for cooking in Chinese adults, particularly prolonged exposure and to some extent wood-burning, was associated with higher risks of chronic liver disease (CLD) mortality, especially liver cancer.
- Tobacco use, particularly among those who started smoking regularly at a young age or who were heavy smokers, was also associated with higher risks of CLD mortality in Chinese adults.
- Individuals who switched from solid to clean fuels, or those who quit smoking not because of illness, had minimal excess risks of CLD mortality, comparable to those of long-term clean fuel users or never-regular smokers.

Over 800 million individuals worldwide are affected by chronic liver diseases (CLD), including liver cirrhosis and liver cancer, with about one-third living in China.^1^^,^^2^ Hepatitis B and C infections have been the main risk factors for CLD in China, but successful hepatitis B vaccination campaigns in recent decades and lifestyle changes have prompted research into other modifiable risk factors.^3^^,^^4^

Smoking has been associated with higher risks of liver disease^5–7^ and more rapid progression of liver cirrhosis or cancer.^8^^,^^9^ Fine particulate matter (PM_2.5_) and toxic organic and inorganic chemicals found in tobacco smoke enter the systemic circulation^10^^,^^11^ and are metabolized in the liver, causing chronic hepatic damage.^12^

Recently several studies have suggested that exposure to air pollutants could also cause liver injury^12–15^ and increased risks of liver disease.^16–18^ Domestic burning of solid fuels (e.g. coal and biomass) for cooking is widely practised by half of the world’s population, and results in high levels of PM_2.5_ and carcinogens in the household environment.^19^ However, no large cohort studies have examined the relevance of solid fuel use for CLD. Indeed, despite accounting for approximately 450 million domestic solid fuel users^20^ and 320 million smokers,^21^ little evidence from China exists on the joint effects of solid fuel use and tobacco smoking for CLD risks.

In a large prospective cohort study of 0.5 million adults in China we investigated: (i) the independent associations of solid fuel and tobacco use on risks of major CLD mortality; and (ii) possible interactions between these exposures in relation to CLD mortality; in urban and rural areas of China.

## Methods

### Study population

This study was approved by the Ethical Review Committee of the Chinese Center for Disease Control and Prevention (Beijing, China) and the Oxford Tropical Research Ethics Committee, University of Oxford (Oxford, UK).

Details of design and methods of the China Kadoorie Biobank (CKB) have been previously reported.^22^^,^^23^ During 2004–08, 512 891 adults aged 30–79 years were recruited from 10 areas across China (Supplementary Figure 1, available as Supplementary data at *IJE* online). At baseline, trained health workers administered an electronic questionnaire to collect information on sociodemographics, lifestyle behaviour (e.g. smoking, drinking), cooking-related exposures and medical history.^22^^,^^23^ All participants had physical measurements recorded, including weight, height, blood pressure and a 10-ml blood sample collected for long-term storage and desktop measurements of random blood glucose and hepatitis B virus surface antigen (HBsAg).^22^^,^^23^ All participants provided written informed consent to the study.

### Assessment of solid fuel use for cooking and of tobacco smoking

Exposure assessment methods for solid fuel and tobacco use have been described in detail elsewhere.^24–26^ Briefly, participants were asked to recall, for up to their three most recent residences, the duration of residence [median (interquartile range): duration 40 (29–50) years, covering ≥ 20 years in 91% of participants], cooking frequency, primary cooking fuel (defined as the fuel type used most frequently and for the longest duration), and cooking stove ventilation (i.e. the presence of chimney or extractor associated with cooking stove(s) used). Participants who reported cooking weekly or daily (‘regular cooking’) were grouped into long-term solid fuel use categories (‘always clean fuels’, ‘solid to clean fuels’ or ‘always solid fuels’) according to their primary fuel use status in each residence. Clean fuels included gas and electricity, whereas solid fuels included wood and coal. Participants who had always used solid fuels were further categorized according to the specific solid fuel types used (‘always coal’, ‘always wood’, ‘mix of coal and wood’) and duration exposed (<20, 20–39, ≥40 years). Duration exposed was derived by summing the total duration of residence where solid fuels were primarily used for cooking, with a weighting of 1.0 for individuals who cooked daily and 0.5 [approximated by the median number of days (3.5) per week] for those who cooked weekly.

For smoking, questions included frequency (current and past: not smoking, occasionally, on most days, daily or almost every day), amount (current and past), age started smoking, age stopped and the key reason for stopping (due to illness or by choice). Participants were classified as never-regular smokers if they reported never or occasionally smoking (<100 cigarettes throughout their lifetime). Among the ex-regular smokers, about 50% (*n* = 18 130) stopped because of illness, which may lead to reverse causality bias. Therefore, they were also analysed separately from those who stopped for other reasons, to better distinguish the potential implications of smoking cessation before onset of disease. A composite exposure of long-term fuel use (always clean fuels or always solid fuels) and smoking status (never- or ever-regular smoking) was derived to investigate the potential joint effects of both exposures.

### Follow-up and outcome definition

Vital status of all participants was checked annually by electronic linkage to the national death registry,^27^ and confirmed with local residential and health insurance records and community administrators. For the small number of deaths (∼5%) that occurred without previous medical attention, standardized verbal autopsies were conducted to determine the most probable cause.^28^ By 1 January 2017 (CKB data release version 15), 44  037 (8.6%) participants had died and 4781 (0.9%) were lost to follow-up. Death events were coded using the International Classification of Diseases, 10th Revision (ICD-10), by trained staff blinded to baseline data. The primary outcome investigated was deaths from major CLD (ICD-10: B18-B19, B94.2, C22, K70, K72-K77) recorded as the underlying cause (Supplementary Table 1, available as Supplementary data at *IJE* online). Participants who died from other causes, were lost to follow-up or did not have an event were censored.

### Statistical analysis

Individuals were excluded if they had: (i) potentially unreliable recall information, indicated by a difference between recalled duration of residence and age greater than 1 year (*n *= 2189); (ii) self-reported physician diagnosis of liver cirrhosis or hepatitis (*n* = 6193); (iii) any cancer (*n* = 2577); (iv) missing data on body mass index (BMI; *n* = 2); or (v) no kitchen at home (*n* = 730), yielding a total of 501 104 participants for the present analyses (Supplementary Figure 2, available as Supplementary data at *IJE* online).

For cooking-related analyses, additional participants were excluded if they: (i) reported using unspecified fuels at any recalled residences (*n* = 3307); (ii) had cooked regularly but stopped at baseline (*n *= 19 210); (iii) had switched from clean to solid fuels (*n* = 988); or (iv) had never cooked regularly (*n* = 128 003), yielding a total of 350 349 participants (Supplementary Figure 2, available as Supplementary data at *IJE* online).

**Figure 1.**
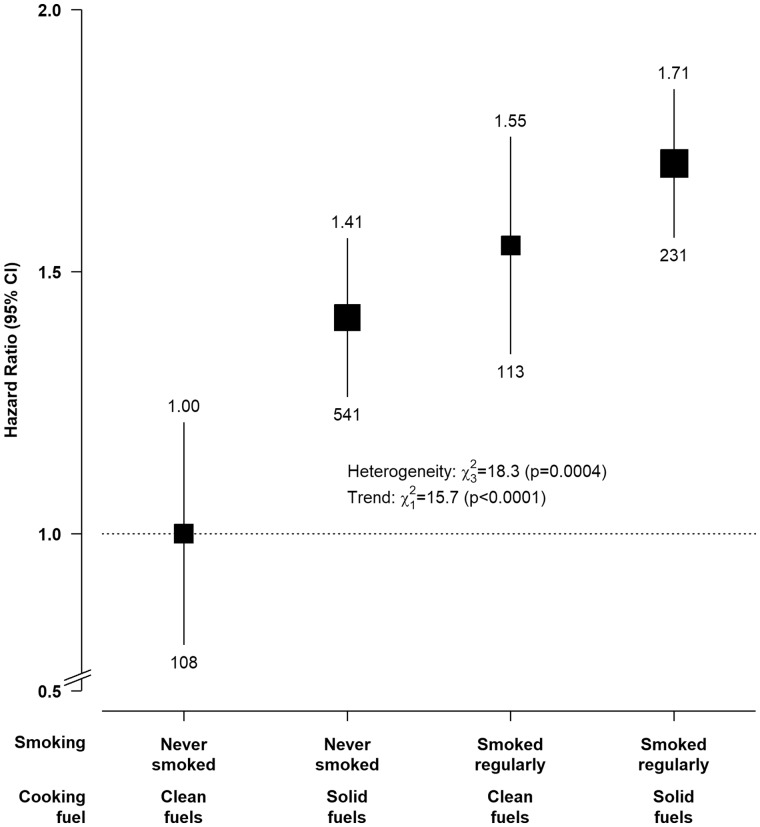
Associations of solid fuel use and tobacco smoking with chronic liver disease mortality. Adjusted hazard ratios were stratified by age-at-risk, sex and study area and adjusted for education, household income, alcohol consumption, long-term heating fuel exposure, cooking stove ventilation, body mass index, prevalent diabetes, hepatitis B surface antigen status and length of recall period. The boxes represent hazard ratios, with the size inversely proportional to the variance of the logarithm of the category-specific log risk [which also determines the confidence interval (CI)], and the vertical lines represent 95% CIs. The numbers above the vertical lines are point estimates for hazard ratios (HRs), and the numbers below the lines are numbers of events. The analysis was restricted to individuals who had data on solid fuel use and smoking (*n* = 350 349).

We used direct standardization to estimate age, sex and study area-adjusted percentages or means of baseline characteristics by long-term cooking fuel and smoking exposure. Stratified Cox regression with time in study as the timescale was used to obtain hazard ratios (HRs) and 95% confidence intervals (CIs) for CLD mortality associated with solid fuel use and tobacco smoking. Models were stratified by age-at-risk (5-year intervals), sex and study area (10 groups) and adjusted for education level (no formal education, primary, middle school, ≥high school), household income (<10 000, 10 000–19 999, 20 000–24 999, ≥25 000 yuan/year), alcohol consumption (never, occasional, ex-regular, current-regular), BMI (continuous), diabetes, HBsAg test result (negative, positive, unclear, missing), length of recall period (continuous), cooking stove ventilation (all, some, none), heating fuel exposure (always clean, solid to clean, always solid, never used heating, others), plus mutual adjustment for cooking fuel exposure and smoking habits. Standard tests using Schoenfeld residuals^29^ and comparisons of the HRs of the first 5 and subsequent years of follow-up for the main cooking and smoking exposures suggested no evidence of violation of the proportional hazard assumption. For graphical representation of the HRs and CLD in Figure 1, the group-specific CIs of HRs were calculated from the variance of the log hazard in each category, to allow comparisons of the HRs of any two categories of exposure and not just with the reference category.^30^ To quantify the potential effect of interaction between smoking and solid fuel use in relation to CLD mortality, the relative excess risk due to interaction (RERI) was calculated as previously described by Li and Chambless.^31^ A positive RERI with 95% CI not overlapping the null indicates the presence of an additive interaction.^31^

A priori subgroup analyses by baseline characteristics were undertaken to investigate potential effect modification, using χ^2^ tests for heterogeneity (interaction) or trend (for ordered subgroups) based on the inverse variance-weighted sum of log(HRs) across strata.^32^ Among the major CLDs examined, liver cancer (ICD-10: C22) is the hallmark of the terminal stages of CLD and accounted for about 75% of all CLD deaths in the study population (Supplementary Table 2, available as Supplementary data at *IJE* online). As a sensitivity analysis, the main analyses were repeated to examine the separate effects on death from liver cancer and other CLDs. Additional sensitivity analyses using sequential adjustment were conducted to investigate the potential impact of residual confounding. Additional covariates adjusted for were: physical activity, systolic blood pressure, self-reported health status, consumption of meat, preserved vegetables, and fresh fruit and leg length (a proxy for early life conditions). The impact of reverse causality was assessed by restricting to HBsAg seronegative participants (*n *= 476 849), excluding participants with poor self-reported health (*n* = 50 876), excluding participants with self-reported or screening-detected diabetes at baseline (*n* = 29 563), and excluding the first 3 years of follow-up. Solid fuels were predominantly used in three (∼97% in Gansu, Henan, and Hunan) of the 10 study areas and rarely used in two areas (<2% in Qingdao and Harbin), in which the numbers of cases were relatively modest and a sensitivity analysis excluding these areas was conducted. All analyses were conducted using SAS software version 9.3.

## Results

### Baseline characteristics

Of the 501 104 participants with no history of liver diseases or any cancer [mean age (SD) 52.0 (10.7) years; 59% women] at baseline, 70% reported cooking regularly at baseline, of whom 49% primarily used solid fuels during the recall period, and 26% were current-regular smokers (61.3% in men; 2.4% in women) (Table 1 and Supplementary Table 3, available as Supplementary data at *IJE* online). Compared with the participants who had always used clean fuels for cooking, those who had always used solid fuels were older, more likely to be women, less educated, had lower household income, smoked or drank less regularly and were more likely to have an unventilated cooking stove or used solid fuels for heating; fewer had diabetes at baseline but more reported poor health. Compared with never-regular smokers, regular smokers were older, less educated, had lower household income, drank more regularly and more reported poor health. Compared with participants included for cooking-related analyses, those excluded (i.e. who reported never cooking regularly) were mostly men, slightly more educated, used solid fuels for heating less often and had a higher prevalence of diabetes and self-reported poor health at baseline (Supplementary Table 3, available as Supplementary data at *IJE* online).

**Table 1.** Baseline characteristics of participants according to long-term primary cooking fuel type and smoking status^a^

	Cooking fuel^b^				Smoking status			
Characteristics	Always clean	Solid to clean	Always solid	Total	Never- regular	Ex- regular	Current- regular	Total
Total participants, *n*	85 350	94 006	170 993	350 349	339 531	29 383	132 190	501 104
Age (SD), years	47.5 (10.1)	52.1 (10.0)	54.7 (10.5)	52.1 (10.5)	51.8 (10.6)	60.6 (10.6)	56.0 (10.4)	52.0 (10.7)
Women, %	50.1	83.5	77.4	76.9	84.5	5.5	5.1	59.2
Urban, %	87.0	82.9	9.8	47.8	47.3	39.4	39.7	44.2
Education level, %								
No formal education	10.7	14.2	22.7	21.7	18.1	20.9	19.1	18.6
Primary	24.6	28.5	36.4	31.5	31.0	33.4	34.1	32.2
Secondary or above	64.7	57.3	40.9	46.8	50.9	45.7	46.9	49.2
Annual household income								
<10, 000 yuan	18.9	20.1	39.0	28.8	27.7	29.1	29.6	28.1
10 000–19 999 yuan	26.4	30.6	31.4	29.2	28.7	30.1	30.1	29.1
≥20 000 yuan	54.7	49.4	29.6	42	43.6	40.8	40.3	42.8
Current-smoker in males, %	58.5	56.7	61.3	61.3	–	–	–	67.7
Current-smoker in females, %	1.9	2.5	3.5	2.4	–	–	–	2.8
Regular drinker in males, %	35.9	39.7	33.0	37.7	21.9	32.2	38.9	2.1
Regular drinker in females, %	2.6	2.1	2.0	2.0	1.8	5.4	9.8	33.6
Always solid fuel for cooking, %	–	–	–	48.8	23.5	30.2	33.9	23.8
No cooking stove ventilation, %	20.0	20.0	30.3	22.8	35.8	21.8	23.9	35.7
Always solid fuel for heating, %^c^	34.4	32.1	63.0	43.2	46.0	47.5	47.7	46.3
Leg length (SD), cm	73.7 (4.8)	73.4 (4.3)	73.1 (4.3)	72.3 (4.5)	73.3 (4.3)	73.5 (4.4)	73.7 (2.7)	73.4 (4.8)
BMI (SD), kg/m^2^	23.9 (3.4)	24.1 (3.4)	23.4 (3.4)	23.8 (3.4)	23.8 (3.4)	24.6 (4.3)	23.5 (2.5)	23.7 (3.4)
BMI at age 25 (SD), kg/m^2^	21.7 (2.5)	22.0 (2.7)	22.1 (2.7)	2.7 (5.3)	21.9 (2.7)	21.8 (4.3)	22.0 (2.4)	21.9 (2.6)
Seropositive HBsAg, %^d^	2.4	2.8	2.9	2.6	2.7	2.0	3.0	2.7
Prevalent diabetes, %	6.8	6.9	5.2	6.1	5.9	9.1	6.8	5.9
Self-reported poor health, %	8.6	9.1	11.5	10.5	9.8	15.2	12.3	10.1

a Means and percentages were adjusted for age, sex and study areas where appropriate.

b Participants who have never cooked regularly (*n* = 128 003) and those who used other unspecified fuels, switched from clean to solid fuels or cooked irregularly (*n *= 23 505) during the recall period were excluded.

c Participants from Haikou (*n* = 28 849) and Zhejiang (*n* = 56 353), <1% of whom reported using heating in winter, were excluded from the denominator.

d HBsAg: hepatitis B virus surface antigen.

### Association of solid fuel use and smoking with major CLD mortality

During 4.3 million person-years of follow-up [median (IQR) 10.1 (9.2 to 11.1) years], 2461 major CLD deaths were recorded. Participants who died from CLD were older, more likely to be men, less educated, had lower income, more likely to smoke and drink at baseline, used solid fuels for cooking (but no difference for heating), had non-negative HBsAg test result, had diabetes, and reported poor health (Supplementary Tables 4 and 5, available as Supplementary data at *IJE* online).

Compared with long-term clean fuel use, solid fuel use for cooking was associated with higher risks of CLD deaths, with adjusted HR of 1.26 (95% CI, 1.02–1.56) (Table 2). In contrast, of individuals who had switched from solid to clean fuels [median (inter-quartile range) years since switching: 14.0 (9.0–19.0)] had no excess risks of CLD (1.07, 0.90–1.29). However, the excess risks associated with long-term solid fuel use were not attenuated by the presence of cooking stove ventilation (Supplementary Table 6, available as Supplementary data at *IJE* online).

**Table 2.** Adjusted hazard ratios for major chronic liver disease mortality by long-term cooking fuel use and smoking habit

	Number of events	Rates (/100 000 person-years)^a^	HR (95% CI)^b^
Long-term cooking fuel			
Always clean (common reference group)	266	35.7	(Reference)
Solid to clean	356	38.3	1.07 (0.90–1.29)
Always solid	837	45.0	1.26 (1.02–1.56)
Duration exposed (mean), years			
<20 (12.4)	289	43.0	1.21 (0.96–1.53)
20–39 (28.0)	328	46.0	1.30 (1.02–1.65)
40+ (48.0)	220	46.7	1.32 (1.01–1.72)
*P*_Trend_			0.023
Types of solid fuels			
Always coal	271	38.9	1.07 (0.81–1.41)
Mix of coal and wood	134	43.3	1.19 (0.90–1.57)
Always wood	432	48.3	1.33 (1.07–1.66)
Smoking			
Never-regular (common reference group)	1148	43.3	(Reference)
Ex-regular	275	53.3	1.23 (1.05–1.44)
Current-regular	1042	55.2	1.28 (1.13–1.44)
Age started smoking (mean), years			
≥25 (31.5)	394	48.0	1.12 (0.97–1.29)
20–24 (21.1)	475	57.3	1.33 (1.16–1.53)
<20 (16.8)	448	59.0	1.37 (1.19–1.58)
*P*_trend_			<0.0001
Daily amount smoked (mean), no. of cigarette			
<15 (7.7)	489	51.2	1.19 (1.04–1.36)
15–24 (19.2)	558	55.3	1.29 (1.12–1.47)
≥25 (35.5)trend	270	61.7	1.44 (1.22–1.69)
* P*_trend_			<0.0001

a Rates were weighted by group-specific hazard ratios.

b Hazard ratios were stratified by age-at-risk, sex and study area and adjusted for education, household income, alcohol consumption, smoking status, long-term cooking and heating fuel exposures, cooking stove ventilation, body mass index, prevalent diabetes, hepatitis B virus surface antigen status and length of recall period, where appropriate.

Among long-term solid fuel users, a dose-response relationship was observed between duration exposed and CLD deaths, with adjusted HRs of 1.21 (0.96–1.53), 1.30 (1.02–1.65) and 1.32 (1.01–1.72) in those exposed for <20, 20–39 and ≥40 years, respectively (*P*_trend_ = 0.023). When stratifying for specific fuel types, the association appeared to be stronger (albeit not statistically significant) for predominant wood users (1.33, 1.07–1.66) than for coal users (1.07, 0.81–1.41, *P*_heterogeneity_=0.066) (Table 2).

Compared with never-regular smokers, the corresponding adjusted HRs for CLD death were 1.28 (1.13–1.44) for current smokers and 1.23 (1.05–1.44) for ex-regular smokers [median (inter-quartile range) years since quitting: 9.8 (3.0–14.0)] (Table 2). The excess risks in ex-regular smokers were observed chiefly in those who stopped because of illness [1.29 (1.06–1.55)] but not in those who stopped for other reasons [1.17 (0.95–1.44)].

Among current-regular smokers, those who started at an earlier age and those who smoked more cigarettes daily had higher risks of CLD deaths (all P_trend_ <0.0001), with adjusted HRs of 1.37 (1.19–1.58) in those who started before the age of 20 years and 1.44 (1.22–1.69) among those who smoked the equivalent of ≥25 cigarettes per day (Table 2).

Figure 1 shows the joint effects of solid fuel use and tobacco smoking on CLD mortality. Compared with never-smoking clean fuel users, regular-smoking solid fuel users had the highest risks (1.71, 1.32–2.20), and the excess risks were lower in regular-smoking clean fuel users (1.55, 1.17–2.06) and never-smoking solid fuel users (1.41, 1.10–1.82). No evidence of additive interaction between solid fuel use and smoking was observed (RERI = 0.11; 95% CI −0.30–0.52).

**Figure 2.**
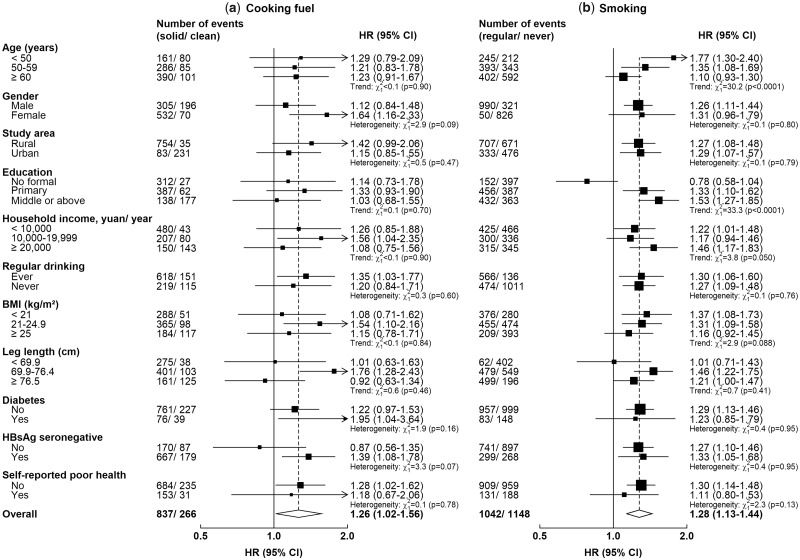
Adjusted hazard ratios for chronic liver disease mortality associated with long-term primary solid fuel use for cooking and regular smoking, stratified by baseline characteristics. Hazard ratios (HRs) were stratified by age-at-risk, sex and study area and adjusted for education, household income, alcohol consumption, smoking habits, long-term cooking and heating fuel exposures, cooking stove ventilation, body mass index, prevalent diabetes, hepatitis B surface antigen (HBsAg) status and length of recall period, where appropriate. The black boxes represent HRs, with the size inversely proportional to the variance of the logarithm of the HR, and the horizontal lines represent 95% CIs. Cooking-related analyses were restricted to 350 349 participants who have cooked regularly throughout their three most recent residences; smoking-related analyses were conducted in 501 104 participants.

Regarding the association between solid fuel use and CLD deaths, there was no evidence of effect modification or trend across all subgroups examined (*P*_heterogeneity_ or *P*_trend_ >0.05 for all comparisons) (Figure 2). The adjusted HR was somewhat stronger in HBsAg seronegative participants (1.39, 1.08–1.78) than in the non-seronegative participants (including positive or uncertain results or missing data; 0.87, 0.56–1.35) (*P*_heterogeneity_ = 0.07). The association of CLD mortality with smoking was stronger in younger participants (*P*_trend_ or *P*_heterogeneity_ <0.0001) and weaker in participants with lower education level (*P*_trend_ or *P*_heterogeneity_ <0.001).

When deaths from liver cancer and other CLDs were analysed separately, the direction and magnitude of association were broadly consistent with the main analyses, albeit with wider CIs, particularly for solid fuel use and other CLD deaths (Supplementary Table 7, available as Supplementary data at *IJE* online). The associations of solid fuel use and smoking with CLD deaths were unaltered in the sensitivity analyses, including sequential adjustments (for physical activity levels, systolic blood pressure, self-reported general health, diet and leg length) and restriction to HBsAg seronegative participants. Exclusion of those with self-reported poor health, diabetes at baseline and the first 3 years of follow-up yielded adjusted HRs for solid fuel use that were considerably stronger but with wider CIs (Table 3). Similarly, the adjusted HR (1.31, 0.88–1.95) associated with solid fuel use remained consistent after excluding the study areas with insufficient number of cases, although the 95%CI included the null.

**Table 3.** Sensitivity analysis for adjusted hazard ratios for major chronic liver disease mortality by long-term cooking fuel use and smoking habit

	Cooking: Always solid versus always clean (*n* = 350 349)			Smoking: Current-smokers versus never-smokers (*n* = 501 104)		
	Number of events	Rate/100 000 person-years^a^	HR (95% CI)	Number of events	Rate/ 100 000 person-years^a^	HR (95% CI)
Main analysis^b^	837	45.0	1.26 (1.02–1.56)	1042	55.2	1.28 (1.09–1.50)
+ additional adjustment^c^	837	45.2	1.28 (1.03–1.59)	1042	55.4	1.28 (1.17–1.40)
+ excluding HBsAg uncertain or missing^d^	805	45.0	1.31 (1.05–1.64)	1011	55.3	1.30 (1.10–1.52)
+ restricting to HBsAg seronegative^d^	667	36.7	1.40 (1.09–1.80)	743	43.4	1.27 (1.06–1.54)
+ excluding self-reported poor health	542	33.9	1.38 (1.05–1.82)	643	40.8	1.29 (1.05–1.58)
+ excluding diabetes at baseline	499	31.5	1.35 (1.01–1.80)	599	38.7	1.30 (1.05–1.61)
+ excluding first 3 years of follow-up	383	35.1	1.39 (1.00–1.94)	454	42.6	1.27 (1.00–1.62)
+ excluding areas with low case numbers^e^	147	32.9	1.31 (0.88–1.95)	–	–	–
Analysis restricting to HBsAg seropositive^b^	138	323.2	0.97 (0.59–1.61)	268	464.9	1.33 (0.95–1.86)

a Rates were weighted by the group-specific hazard ratios.

b Hazard ratios were stratified by age-at-risk, sex and study area and adjusted for education, household income, alcohol consumption, smoking status, long-term cooking and heating fuel exposures, cooking stove ventilation, body mass index, prevalent diabetes, HBsAg status and length of recall period, where appropriate.

c Hazard ratios were further adjusted for physical activity levels, systolic blood pressure, self-reported health, diet (consumption of meat, preserved vegetables and fresh fruit), and leg length.

d HBsAg: hepatitis B virus surface antigen.

e Excluding Qingdao and Harbin, two urban areas where few people consistently used solid fuels long term, and Gansu, Henan and Hunan, three areas where few participants consistently used clean fuels long term.

## Discussion

This large prospective study demonstrated that long-term solid fuel use for cooking and smoking were each independently associated with higher risks of CLD mortality. We observed higher risks among those with a longer duration [≥20 years of solid fuel use or who started smoking earlier (by 20 years of age)] and higher levels of exposure (persistent use of wood or smoked a greater amount of cigarettes).

Historically, exposure to household air pollution from solid fuel use was believed to affect mainly the respiratory and circulatory systems,^33^ although recent evidence suggests that the systemic effects of air pollutants^34^ also affect the liver. Animal studies have demonstrated that particulate air pollution could potentially induce liver damage by both direct toxicity and systemic pro-inflammatory effects.^12–14^ Recent epidemiological studies have also reported associations of ambient PM_2.5_ exposure with impaired liver function,^15^^,^^16^ excess risks of developing liver cancer^16–18^ and poor prognosis.^35^ However data from such studies, with relatively low PM_2.5_ levels (median or mean 11.3 to 36.0  μg/m^3^),^15–18^^,^^35^ were unable to demonstrate a dose-response relationship between PM_2.5_ exposure and CLD mortality, especially at higher levels related to solid fuel use (300 to 1500  μg/m^3^) or smoking (1000–10 000  μg/m^3^).^10^^,^^11^^,^^36^^,^^37^ For comparison, a supralinear exposure-response function (levelling off at high PM_2.5_ concentrations) has been used by the Global Burden of Disease, Injuries and Risk Factors Study (GBD) to estimate the risk of cardiovascular death, by combining studies with different sources of PM_2.5_ (ambient air pollution, passive and active smoking).^38^ In contrast with previous studies, the stronger associations of solid fuel and tobacco use with CLD death observed in the present study provides important new evidence on the shape of the dose-response relationship of PM_2.5_ exposure and CLD mortality.

In subgroup analyses, the association of solid fuel use with CLD death was somewhat stronger in HBsAg seronegative participants [HR, 1.39 (95% CI, 1.08–1.78)], although the difference observed should be interpreted with caution due to the small number of cases in the non-seronegative subgroup [0.87 (0.56–1.35)]. Although the play of chance cannot be fully excluded, analysis also suggests that the additional risks associated with solid fuel use could be negligible among those who have hepatitis B virus infection, which is one key risk factor for CLD mortality [HBsAg seropositive versus seronegative: HR, 13.1 (95% CI, 11.8–14.4); Supplementary Table 8, available as Supplementary data at *IJE* online]. Similar findings were observed in participants who had a previous of diagnosis of liver cirrhosis or hepatitis at baseline (who were excluded from all main analyses) (Supplementary Table 9, available as Supplementary data at *IJE* online).

Smoking is an established cause of liver cancer^39^ and has also been associated with the development and accelerated progression of liver diseases.^5–9^ In the present study, the excess risks of CLD death were observed in regular smokers, with stronger associations among younger participants but weaker in those with lower attained education. The excess relative risks of CLD death associated with smoking observed in the present study were somewhat smaller than those observed in Western high-income countries (HR range 1.5 to 3.4).^40^^,^^41^ This could be because the tobacco epidemic has already reached its peak in Western countries, whereas the ‘full effect’ of the impact of smoking is yet to be observed in China.^40^ Starting smoking earlier has been shown to be associated with higher risks of cardiovascular mortality.^25^ The findings of the present study reinforce the need for strong tobacco control measures, and also may have implications for the planning of future health care service provision in China and elsewhere.^42^

The present study demonstrated that solid fuel and tobacco use were each independently associated with CLD risk, with smoking [HR (95% CI) 1.55 (1.17–2.06)] appearing to confer higher risks than solid fuel use [1.41 (1.10–1.82)], compared with never-smoking clean fuel users. Importantly, concomitant exposure was associated with the largest excess risks [1.71 (1.32–2.20)], even though PM_2.5_ level in cigarette smoke could be an order of magnitude higher than solid fuel smoke. This may be particularly relevant to many low- and middle-income countries where the tobacco industry has targeted females who are often already exposed to household air pollution due to their societal gender role.^43–45^

The chief strengths of the present study included the large sample size and prospective design, and the novel investigation of the association between solid fuel use and CLD deaths. We also were able to separate ex-smokers who stopped because of illness from those who stopped for other reasons, thus highlighting the benefits of cessation on health. However, the study also had several limitations. First, we do not have data on some potential confounders such as exposure to aflatoxin, herbal medicines or other environmental contaminants (e.g. arsenic in water).^3^ Among these, aflatoxin, a hepatoxic fungal metabolite and one of the risk factors for CLD, is strongly linked to poverty and is highly region-specific in China (with higher levels in the hot and humid south).^46^ Two CKB study areas (Liuzhou of Guangxi and Suzhou of Jiangsu Provinces), which traditionally have had high exposure to aflatoxin, are urban cities where clean fuels are predominantly used. In an attempt to minimize the impact of unmeasured confounding, all analyses were stratified by study area and adjusted for baseline HBsAg status and socioeconomic and lifestyle indicators (education, income and diet). We further excluded participants who were not seronegative and those reporting poor health at baseline in the sensitivity analyses, and did not find material changes in the associations. Second, whereas CLD deaths were ascertained from well-established death registries, under-diagnosis of liver diseases is common in China.^3^ Such under-diagnosis is likely to be differential and linked to lower socioeconomic status or residence in rural areas, both of which are strongly associated with solid fuel use and potentially biasing the association towards the null. Third, data on tobacco and solid fuel use were self-reported and the lack of data on secondary fuel use may have diluted the observed associations. However, we endeavoured to refine our assessment of solid fuel exposure by incorporating personal cooking frequency and excluding those who reported that they did not cook regularly.^26^ Fourth, liver disease and the exposures of interest, particularly solid fuel use, are closely linked to socioeconomic status. Despite the efforts made in the analyses, residual confounding from socioeconomic status cannot be fully excluded. Finally, we focused on CLD deaths rather than cause-specific subtypes, because of the dominance of liver cancer (contributing ∼75% of all CLD deaths) in the present study and this limits the generalizability of our findings. Furthermore, individuals with CLD may be susceptible to competing risks of cardiovascular death,^47^ although there were only 127 deaths with non-CLD as the underlying cause where CLD had been listed as an associated cause on the death certificate. The inclusion of such cases did not materially alter the results (data not shown).

The present study provides important new evidence linking solid fuel use and smoking with liver disease mortality in a Chinese population. Without an accelerated shift to cleaner domestic energy and stringent tobacco control measures, the double burden of household air pollution and tobacco use in low- and middle-income countries is likely to result in an increasing burden of liver disease mortality, despite the implementation of effective hepatitis B vaccination campaigns.

## Funding

The present study was supported by the UK Medical Research Council: Global Challenges Research Fund – Foundation Award (MR/P025080/1). K.H.C. is a recipient of the Early Career Research Fellowship from the Nuffield Department of Population Health, University of Oxford. The CKB baseline survey and the first re-survey were supported by the Kadoorie Charitable Foundation in Hong Kong. The long-term follow-up has been supported by the UK Wellcome Trust (202922/Z/16/Z, 104085/Z/14/Z, 088158/Z/09/Z) and grants from the National Natural Science Foundation of China (918433302) and from the National Key Research and Development Program of China (2016YFC0900500, 2016YFC0900501, 2016YFC0900504, 2016YFC1303904). The British Heart Foundation, UK Medical Research Council and Cancer Research provide core funding to the Clinical Trial Service Unit and Epidemiological Studies Unit at Oxford University for the project. The funders had no role in the study design, data collection, data analysis and interpretation, writing of the report or decision to submit the article for publication.

## Supplementary Material

dyz216_Supplementary_Data
