# Supplementary material for: Solid fuels for cooking and tobacco use and risk of major chronic liver disease mortality: a prospective cohort study of 0.5 million Chinese adults
Source: Int J Epidemiol. 2019 Oct 25;49(1):45–55. doi: 10.1093/ije/dyz216 (PMC7124491; doi:10.1093/ije/dyz216)
Supplement: dyz216_Supplementary_Data [file dyz216_supplementary_data.docx]

**Supplementary Materials**

**Solid Fuels for Cooking and Tobacco Use and Risk of Major Chronic Liver Disease Mortality: a Prospective Cohort Study of 0.5 million Chinese Adults**

Ka Hung Chan, Derrick A Bennett*, Om P Kurmi, Ling Yang, Yiping Chen, Jun Lv, Yu Guo, Zheng Bian, Canqing Yu, Xiaofang Chen, Caixia Dong, Liming Li, Zhengming Chen, Kin Bong Hubert Lam on behalf of the China Kadoorie Biobank Study Group^†^

* Corresponding author

† The members of the China Kadoorie Biobank Collaborative Group and its steering committee are listed in the Appendix

**Table of Contents**

[**Supplementary Table 1.**](#ST1) Details of major chronic liver disease defined by ICD-10 codes ……………………………………………………………………………………………p.3

[**Supplementary Table 2.**](#ST3) Number of deaths attributed to specific chronic liver diseases (CLD) ……………………………………………………………………………......................p.4

[**Supplementary Table 3.**](#ST2) Baseline characteristics of participants according to long-term cooking behaviour……………………………………………………………………………...p.5

[**Supplementary Table 4.**](#ST4) Baseline characteristics according to major chronic liver disease mortality……………………………………………………………………………...................p.6

[**Supplementary Table 5.**](#ST5) Distribution and rates of major chronic liver disease mortality according to age, sex and study area. ……………………………………..........................p.7

[**Supplementary Table 6.**](#ST6) Adjusted hazard ratios for chronic liver disease mortality by long-term cooking fuel use and cookstove ventilation exposure…………………….................p.8

[**Supplementary Table 7.**](#ST72) Adjusted hazard ratios for liver cancer and other chronic liver disease mortality by long-term cooking fuel use and smoking habit………………………p.9

[**Supplementary Table 8.**](#ST7) Adjusted hazard ratios for major chronic liver disease mortality according to baseline HBsAg test results…………………………………..........................p.10

[**Supplementary Table 9.**](#ST8) Adjusted hazard ratios for chronic liver disease mortality by long-term cooking fuel use and smoking habit in participants with a history of liver cirrhosis or hepatitis at baseline (N = 6116) ………………………………….......................................p.11

[**Supplement Figure 1.**](#SF1) Geographic location of study sites of the China Kadoorie Biobank…………………………………………………………………………………………..p.12

[**Supplementary Figure 2.**](#SF2) Participant exclusion……………………………………….......p.13

[**References for the supplementary material**](#RF)………………………………….................p.14

| **Supplementary Table 1. Details of major chronic liver disease defined by ICD-10 codes*** | |
| --- | --- |
| **ICD-10 Code** | **ICD-10 description** |
| B18 | Chronic hepatitis |
| B19 | Unspecified hepatitis |
| B94.2 | Sequelae of viral hepatitis |
| C22 | Malignant neoplasm of liver and intrahepatic bile ducts |
| K70 | Alcoholic liver disease |
| K72 | Hepatic failure, not elsewhere classified |
| K73 | Chronic hepatitis, not elsewhere classified |
| K74 | Fibrosis and cirrhosis of liver |
| K75 | Other inflammatory liver diseases |
| K76 | Other disease of liver |
| K77 | Liver disorders in diseases classified elsewhere |

* In the present study, chronic liver disease death is defined as any death with any of the enlisted ICD-10 codes as the underlying cause

| **Supplementary Table 2. Number of deaths attributed to specific chronic liver diseases (CLD)** | | |
| --- | --- | --- |
| **Specific causes of CLD death** | **ICD-10 code** | **Number of deaths** |
| Liver cancer | C22 | 1855 |
| Liver cirrhosis and fibrosis | K70.2, K70.3, K74 | 309 |
| Chronic hepatitis | B18 | 211 |
| Alcoholic liver disease | K70 | 63 |
| Non-alcoholic non-viral inflammatory liver disease | K75 | 13 |
| Non-inflammatory liver disease | K76 | 9 |

| **Supplementary Table 3. Baseline characteristics of participants according to long-term cooking behaviour*** | | | | | |
| --- | --- | --- | --- | --- | --- |
|  | **Included in the analyses** |  | **Excluded from the analyses^†^** | | **Total** |
| **Characteristics** | **Regular cooks** |  | **Never regular cooks** | **Others** |  |
| **Total participants, N** | 350,349 |  | 127,542 | 23,213 | 501,104 |
| **Age (SD), years** | 52.4 (10.5) |  | 48.9 (11.0) | 53.1 (11.2) | 52.0 (10.7) |
| **Women, %** | 77.5 |  | 11.8 | 60.9 | 59.2 |
| **Education level, %** |  |  |  |  |  |
| No formal education | 18.8 |  | 16.7 | 17.3 | 18.6 |
| Primary | 32.3 |  | 28.8 | 29.9 | 32.2 |
| Secondary or above | 48.9 |  | 54.5 | 52.8 | 49.2 |
| **Annual household income** |  |  |  |  |  |
| < 10,000 yuan | 29.8 |  | 23.9 | 23.9 | 28.1 |
| 10,000-19,999 yuan | 28.6 |  | 28.3 | 29.6 | 29.1 |
| ≥ 20,000 yuan | 41.6 |  | 47.8 | 46.5 | 42.8 |
| **Regular smoker in male, %** | 59.9 |  | 62.3 | 60.2 | 61.3 |
| **Regular smoker in female, %** | 2.3 |  | 2.5 | 2.7 | 2.4 |
| **Regular drinker in male, %** | 34.0 |  | 33.2 | 33.7 | 2.1 |
| **Regular drinker in female, %** | 2.0 |  | 2.9 | 2.7 | 33.6 |
| **No cookstove ventilated, %** | 24.2 |  | 23.4 | 23.5 | 35.7 |
| **Always solid fuel for heating, %^‡^** | 46.0 |  | 45.0 | 43.0 | 46.3 |
| **Leg length (SD), cm** | 73.3 (4.5) |  | 73.5 (4.3) | 73.4 (4.7) | 73.4 (4.8) |
| **BMI (SD), kg/m^2^** | 23.7 (3.4) |  | 23.7 (3.2) | 23.8 (3.4) | 23.7 (3.4) |
| **BMI at age 25 (SD), kg/m^2^** | 21.9 (2.7) |  | 21.8 (2.4) | 22.0 (2.6) | 21.9 (2.6) |
| **Seropositive HBsAg, %^§^** | 2.7 |  | 2.7 | 2.5 | 2.7 |
| **Prevalent diabetes, %** | 5.8 |  | 7.0 | 7.3 | 5.9 |
| **Self-reported poor health, %** | 10.2 |  | 11.1 | 12.9 | 10.1 |

* Means and percentages were adjusted for age, sex and study area where appropriate.

^†^ Participants who have never cooked regularly (n = 128,003) and those who used other unspecified fuels, switched from clean to solid fuels, or cooked irregularly (n = 23,505) during the recall period were excluded.

**^‡^** Percentages shown were calculated among those who had always have heating during their recall period.

^§^ HBsAg: hepatitis B surface antigen.

| **Supplementary Table 4. Baseline characteristics according to major chronic liver disease mortality*** | | | |
| --- | --- | --- | --- |
|  | **Major chronic liver disease mortality** | |  |
|  | **No** | **Yes** | **All participants** |
| **Total participants, N** | 498,643 | 2,461 | 501,104 |
| **Age (SD), years** | 52.0 (10.6) | 59.3 (9.9) | 52.0 |
| **Female sex, %** | 59.3 | 33.9 | 59.2 |
| **Study area, %** |  |  |  |
| Qingdao | 7.0 | 5.3 | 7.0 |
| Harbin | 11.2 | 8.0 | 11.2 |
| Haikou | 5.7 | 6.4 | 5.7 |
| Suzhou | 10.5 | 5.9 | 10.5 |
| Liuzhou | 9.9 | 7.3 | 9.8 |
| Sichuan | 10.8 | 15.2 | 10.9 |
| Gansu | 9.6 | 15.8 | 9.6 |
| Henan | 12.4 | 14.9 | 12.4 |
| Zhejiang | 11.2 | 9.0 | 11.2 |
| Hunan | 11.6 | 12.1 | 11.6 |
| **Education level, %** |  |  |  |
| No formal education | 18.6 | 20.8 | 18.6 |
| Primary | 32.2 | 35.4 | 32.2 |
| Secondary or above | 49.2 | 43.8 | 49.2 |
| **Annual household income** |  |  |  |
| < 10,000 yuan | 28.1 | 33.3 | 28.1 |
| 10,000-19,999 yuan | 29.1 | 30.9 | 29.1 |
| ≥ 20,000 yuan | 42.8 | 35.8 | 42.8 |
| **Current-smoker in male, %** | 61.2 | 63.6 | 61.3 |
| **Current-smoker in female, %** | 2.4 | 4.3 | 2.4 |
| **Current drinker in male, %** | 33.5 | 36.9 | 33.6 |
| **Current drinker in female, %** | 2.1 | 2.6 | 2.1 |
| **Always solid fuels for cooking, %** | 35.7 | 39.9 | 35.7 |
| **No cookstove ventilated, %** | 23.8 | 25.2 | 23.8 |
| **Always solid fuel for heating, %** | 46.3 | 47.8 | 46.3 |
| **Leg length (SD), cm** | 73.4 (4.8) | 73.3 (4.9) | 73.4 |
| **BMI (SD), kg/m^2^** | 23.7 (3.4) | 23.4 (3.5) | 23.7 |
| **BMI at age 25 (SD), kg/m^2^** | 21.9 (2.6) | 22.1 (2.7) | 21.9 |
| **Seropositive HBsAg, %**^†^ | 2.6 | 25.0 | 2.7 |
| **Prevalent diabetes, %^‡^** | 5.9 | 10.3 | 5.9 |
| **Self-reported poor health, %** | 10.1 | 13.8 | 10.1 |

* Means and percentages were adjusted for age, sex and study area where appropriate.

^†^ HBsAg: hepatitis B virus surface antigen.

**^‡^** Prevalent diabetes: self-reported prior doctor diagnosis or screen-detected by random blood glucose test.

| **Supplementary Table 5. Distribution and rates of major chronic liver disease mortality according to age, sex and study area.** | | |
| --- | --- | --- |
|  | **No. of events** | **Rate (no./100,000 person-years) *** |
| **Age, years (mean)** |  |  |
| 30-39 (37.3) | 88 | 11.3 |
| 40-49 (44.8) | 398 | 26.0 |
| 50-59 (54.6) | 806 | 52.1 |
| 60-69 (64.7) | 801 | 94.2 |
| ≥ 70 (72.6) | 368 | 132.7 |
| **Sex** |  |  |
| Male | 1564 | 78.4 |
| Female | 897 | 30.1 |
| **Study area (from North to South)** |  |  |
| Harbin | 232 | 42.2 |
| Qingdao | 147 | 41.5 |
| Gansu | 314 | 65.6 |
| Henan | 322 | 51.9 |
| Suzhou | 203 | 38.9 |
| Sichuan | 372 | 69.0 |
| Zhejiang | 230 | 39.8 |
| Hunan | 287 | 49.6 |
| Liuzhou | 212 | 44.2 |
| Haikou | 142 | 51.1 |

* Rates were weighted, as per previous work,(1) by the group-specific hazard ratios from Cox regression model stratified for age-at-risk, sex, and study area and adjusted for education, household income, alcohol consumption, smoking status, long-term cooking and heating fuel exposures, cookstove ventilation, body mass index, prevalent diabetes, HBAgs status, and length of recall period, where appropriate.

| **Supplementary Table 6. Adjusted hazard ratios for chronic liver disease mortality by long-term cooking fuel use and cookstove ventilation exposure*** | | | |
| --- | --- | --- | --- |
|  | **Number of events** | **Rates^†^** | **HR (95% CI)^‡^** |
| **LONG-TERM COOKING FUEL** |  |  |  |
| Always clean (reference) | 314 | 50.2 | 1.00 (0.88-1.14) |
| Solid to clean | 391 | 51.3 | 1.02 (0.90-1.16) |
| Always solid with ≥ 50% recall period with cookstove ventilation | 558 | 63.6 | 1.27 (1.14-1.41) |
| Always solid with <50% recall period with cookstove ventilation | 362 | 62.8 | 1.25 (1.10-1.43) |

* Cookstove ventilation: the presence of chimney or extractor associated with cookstove(s) being used.
^†^ Rates (per 100,000 person-year) were weighted by group-specific hazard ratios.
^‡^ Hazard ratios were stratified by age-at-risk, sex, and study area and adjusted for education, household income, alcohol consumption, smoking status, long-term heating fuel exposures, body mass index, prevalent diabetes, HBAgs status, and length of recall period.

| **Supplementary Table 7. Adjusted hazard ratios for liver cancer and other chronic liver disease mortality by long-term cooking fuel use and smoking habit** | | | | | | | |
| --- | --- | --- | --- | --- | --- | --- | --- |
|  | **Liver cancer** | | |  | **Other chronic liver disease** | | |
|  | **Number of deaths** | **Rates (/100,000 person-years)*** | **HR (95% CI)^†^** |  | **Number of deaths** | **Rates (/100,000 person-years)*** | **HR (95% CI)^†^** |
| **LONG-TERM COOKING FUEL** |  |  |  |  |  |  |  |
| Always clean (common reference group) | 213 | 27.9 | (Reference) |  | 53 | 7.7 | (Reference) |
| Solid to clean | 267 | 28.1 | 1.01 (0.82-1.24) |  | 89 | 10.4 | 1.36 (0.92-2.00) |
| Always solid | 616 | 33.9 | 1.22 (0.95-1.55) |  | 221 | 11.0 | 1.44 (0.92-2.24) |
| **Types of solid fuels** |  |  |  |  |  |  |  |
| Always coal | 214 | 32.3 | 1.15 (0.83-1.58) |  | 57 | 7.2 | 0.94 (0.54-1.65) |
| Mix of coal and wood | 93 | 31.9 | 1.13 (0.81-1.58) |  | 41 | 11.1 | 1.45 (0.85-2.50) |
| Always wood | 309 | 35.1 | 1.24 (0.97-1.60) |  | 123 | 12.7 | 1.65 (1.04-2.62) |
| **Duration exposed (mean), years** |  |  |  |  |  |  |  |
| <20 (12.4) | 267 | 28.1 | 1.01 (0.82-1.24) |  | 89 | 10.2 | 1.37 (0.93-2.01) |
| 20-39 (28.0) | 220 | 33.5 | 1.21 (0.93-1.57) |  | 69 | 9.5 | 1.26 (0.78-2.05) |
| 40+ (48.0) | 233 | 33.2 | 1.19 (0.91-1.57) |  | 95 | 12.5 | 1.67 (1.03-2.70) |
| P_Trend_ |  |  | 0.0305 |  |  |  | 0.0685 |
| **SMOKING** |  |  |  |  |  |  |  |
| Never-regular (common reference group) | 868 | 33.4 | (Reference) |  | 279 | 9.9 | (Reference) |
| Ex-regular | 214 | 40.3 | 1.21 (1.01-1.44) |  | 60 | 12.8 | 1.29 (0.93-1.79) |
| Current-regular | 773 | 40.8 | 1.22 (1.07-1.41) |  | 267 | 14.4 | 1.45 (1.13-1.86) |
| **Age started smoking (mean), years** |  |  |  |  |  |  |  |
| ≥25 (31.5) | 290 | 35.0 | 1.06 (0.90-1.25) |  | 104 | 13.0 | 1.31 (0.99-1.74) |
| 20-24 (21.1) | 365 | 43.2 | 1.31 (1.12-1.53) |  | 108 | 14.0 | 1.42 (1.06-1.89) |
| <20 (16.8) | 332 | 43.7 | 1.32 (1.13-1.56) |  | 115 | 15.2 | 1.53 (1.15-2.04) |
| P_Trend_ |  |  | <0.0001 |  |  |  | 0.0036 |
| **Daily amount smoked (mean), no. of cigarette** |  |  |  |  |  |  |  |
| <15 (7.7) | 360 | 37.2 | 1.13 (0.97-1.32) |  | 128 | 13.9 | 1.40 (1.07-1.83) |
| 15-24 (19.2) | 424 | 41.1 | 1.25 (1.07-1.45) |  | 134 | 14.1 | 1.42 (1.08-1.88) |
| ≥25 (35.5) | 203 | 47.4 | 1.44 (1.19-1.73) |  | 65 | 14.2 | 1.43 (1.02-2.01) |
| P_Trend_ |  |  | <0.0001 |  |  |  | 0.0262 |

* Rates were weighted by group-specific hazard ratios.
^†^ Hazard ratios were stratified by age-at-risk, sex, and study area and adjusted for education, household income, alcohol consumption, smoking status, long-term cooking and heating fuel exposures, cookstove ventilation, body mass index, prevalent diabetes, HBsAg status, and length of recall period, where appropriate.

| **Supplement Table 8. Adjusted hazard ratios for major chronic liver disease mortality according to baseline HBsAg test results*** | | | |
| --- | --- | --- | --- |
| **HBsAg test results** | **Number of events** | **Rates (no. /100,000 person-years)^†^** | **HR (95% CI)^‡^** |
| Negative | 1834 | 13.4 | Reference |
| Positive | 544 | 174.5 | 13.1 (11.8-14.4) |
| Uncertain | 38 | 40.1 | 3.00 (2.16-4.15) |
| Missing data/ no result | 45 | 15.8 | 1.19 (0.87-1.62) |
| *HBsAg: hepatitis B virus surface antigen. ^†^ Rates were weighted by group-specific hazard ratios. ^‡^ Hazard ratios were stratified by age-at-risk, sex, and study area and adjusted for education, household income, alcohol consumption, smoking status, long-term cooking and heating fuel exposures, cookstove ventilation, body mass index, prevalent diabetes, and length of recall period. | | | |

| **Supplementary Table 9. Adjusted hazard ratios for chronic liver disease mortality by long-term cooking fuel use and smoking habit in participants with a history of liver cirrhosis or hepatitis at baseline (N = 6116)*** | | | |
| --- | --- | --- | --- |
|  | **Number of events** | **Rate/ 100,000 person-years^†^** | **HR (95% CI)^‡^** |
| **LONG-TERM COOKING FUEL** |  |  |  |
| Always clean | 48 | 377.6 | Reference |
| Solid to clean | 35 | 252.4 | 0.67 (0.40-1.12) |
| Always solid | 85 | 611.3 | 1.62 (0.87-3.00) |
| **SMOKING** |  |  |  |
| Never-regular | 119 | 547.8 | Reference |
| Ex-regular | 62 | 597.4 | 1.09 (0.75-1.57) |
| Current-regular | 155 | 564.5 | 1.03 (0.76-1.39) |

* Individuals with self-reported physician diagnosis of liver cirrhosis or hepatitis at baseline, excluding those with prior diagnosis of cancer, missing data on body mass index or potentially unreliable recall information.
^†^ Rates were weighted by group-specific hazard ratios.
^‡^ Hazard ratios were stratified by age-at-risk, sex, and study area and adjusted for education, household income, alcohol consumption, smoking status, long-term cooking and heating fuel exposures, cookstove ventilation, body mass index, prevalent diabetes, HBsAg status, and length of recall period, where appropriate.

**Supplementary Figure 1. Geographic locations of study sites of the China Kadoorie Biobank***

* Adopted from Chen et al. 2010.(2) Solid circles (●) are rural areas and open circles (○) are urban areas included in the China Kadoorie Biobank Study. Number of participant at baseline in each study area is shown in brackets.

**Supplementary Figure 2. Participant exclusion**

*For the main smoking-related analyses.

**References for the supplementary material**

1. Liu X, Bragg F, Yang L, Kartsonaki C, Guo Y, Du H, et al. Smoking and smoking cessation in relation to risk of diabetes in Chinese men and women: a 9-year prospective study of 0·5 million people. Lancet Public Health. 2018;3(4):e167-e76.

2. Chen Z, Chen J, Collins R, Guo Y, Peto R, Wu F, et al. China Kadoorie Biobank of 0.5 million people: survey methods, baseline characteristics and long-term follow-up. Int J Epidemiol. 2011;40(6):1652-66.
